# Perceived needs and level of satisfaction with care by family members of critically ill patients at Muhimbili National hospital intensive care units, Tanzania

**DOI:** 10.1186/s12912-016-0139-5

**Published:** 2016-03-09

**Authors:** Thecla W. Kohi, Marwa W. Obogo, Lilian T. Mselle

**Affiliations:** Department of Nursing and Management, School of Nursing, Muhimbili University of Health and Allied Sciences, P.O. Box 65004, Dar es Salaam, Tanzania; Emergency Department, Muhimbili National Hospital, P.O. Box 65000, Dar es Salaam, Tanzania; Department of Clinical Nursing, School of Nursing, Muhimbili University of Health and Allied Sciences, P. O. Box 65004, Dar es Salaam, Tanzania

**Keywords:** Critically-ill patients, Intensive care unit, Needs of family members, Critical care nurses, Tanzania

## Abstract

**Background:**

Earlier studies in developed and a few developing countries have documented experiences of family members with critically-ill patients. However, in Tanzania no documented studies could be found in this study area. The aim of this study was therefore to explore the Tanzanian family members’ perceived needs and level of satisfaction with care of their critically-ill patients, in the intensive care units in the Muhimbili National Hospital.

**Methods:**

A descriptive cross-sectional study was undertaken, using a quantitative approach. A semi-structured questionnaire was used to collect data. The sample size was 110 respondents, comprised of individuals who visited critically-ill patients in the intensive care unit. Data were analyzed using SPSS Version 14.

**Results:**

The study revealed that 72 % of the family members perceived having a specific person to call at the hospital when a related family member was not available at the hospital as a very important need. Only 23 % of the respondents perceived the need of talking about the possibility of their patients’ death as very important. The nurses’ provision of care to the patients of the family members was found to be satisfactory.

**Conclusion:**

The perceived needs and level of satisfaction of family members of critically-ill patients calls for nurses to improve the quality of care to patients’ family members, which in return will enhance the patient’s recovery.

**Electronic supplementary material:**

The online version of this article (doi:10.1186/s12912-016-0139-5) contains supplementary material, which is available to authorized users.

## Background

Critical illness often comes without warning, leaving little time for patients and their family members to prepare [1]. In the past, critical care nursing was concerned primarily with individual patients, focusing intentionally on the patient, while inclusion of the family system was ignored [1, 2]. According to Van [3], in the critical care environment, family members often serve as the spokespersons when the patient is physiologically and psychologically compromised. Also, because critical illness occurs without warning, family members may feel vulnerable and helpless with no clear knowledge of what to expect from the health care system regarding illness or prognosis [3]. Needs of the family of critically-ill patients are requirements that if not met, become a demand that may produce distress in family members. In developed countries these needs have been addressed according to several studies [1, 2, 4, 5] and have raised a growing awareness to health care providers that attending the needs of these family members is a responsibility that no health care facility can ignore. Family members serve as a bridge between unresponsive patients and health care providers [3]; however, family members are also vulnerable to stress, anxiety and helplessness when their relative is admitted to an Intensive Care Unit (ICU), particularly if their own needs are not met. Thus, these findings underline the importance of assessing the needs that are perceived by family members, to be essential during care of their loved ones in intensive care, in the Africa region, particularly in Tanzania.

Satisfaction with care is related to quality of care and provision of health care facilities as the means of improving care [6, 7]. In developed countries the number of patients who are admitted annually is high. In the United States of America (USA) more than five million patients are admitted to ICUs annually [7]. This implies that the number of family members with different perceived needs and satisfaction are also high.

The annual admission at Muhimbili National Hospital (MNH) in 2009/2010 was 422 patients in the general ICU, and 25 in the cardiac ICU [8]. The top ten conditions leading to admission in the general ICU were peritonitis, ameloblastoma, maltinodular, goiter, hypertensive stroke, cardiogenic shock, tetanus, diabetic mellitus, eclampsia, acalasia of esophagus and renal failure. In the cardiac ICU, admissions were mainly due to valvur replacement or valvectomy, patent ductus arteriosus, mitral valve repair, ventricular septal repair and atria septal repair. With the trend of motorcycles becoming a popular means of transport in the country, the number of motor traffic accidents is alarming. This study did not go in-depth to explore matters related to motor traffic accidents.

The challenge for the nurse working in critical care is to provide care to the critically-ill patients, while at the same time attending to the needs of patients’ family members [9]. This is contributed by the shortage of trained health workers including nurses in Tanzania. Van [3] says that the nurse family-relationship in critical care setting is very important, especially if a family is compromised by their patient’s illness. Usually, it is the family member who knows the history of the unresponsive patient for example, social allergies and medical history [3].

Maxwell et al. [9] reported that 75 % of all patients were unable to participate at the time when difficult decisions about the goals of treatment were to be made [9]. Thus, nurses and doctors must rely on family members to speak on behalf of the patient, give consent for complicated treatment and procedures, or approve the termination of life support efforts. If holistic care is to be practiced, the family needs must be considered together with those of the patient.

The needs of families of critically-ill patients have been addressed in several studies, and nurses’ and families’ perceptions about the needs of these families have been found to differ [4, 10, 11]. The family needs included support, comfort, information, proximity and assurance [10]. In a phenomenological study, the critical care families identified qualities like informing, enhancing, touching and spiriting as the perceived caring nursing behaviours [12]. In some cases, the needs of family members tended to be underestimated by ICU nurses in some aspects, for example, receiving some kind of information, feeling accepted by staff of being informed of changes in the patient’s condition [2, 13]. Other studies have found out that the worst situation for close relatives in ICU is when they do not receive enough information about the critically ill patient’s condition and prognosis [14, 15]. The nurses admitted that they gave priority to the welfare of their loved ones in ICU, and in their very busy state they don’t get around to paying enough attention to family members [13]. This means nurses have to be knowledgeable about the individual needs of both relatives and patients to be able to provide care and support for both [16]. Brown et al. [17] acknowledge that as critical care nurses advocate for critically ill patients, serves as the eyes, ears and voice of those who are at risk

So far, in Tanzania, there is no documented study on the needs and level of satisfaction of families of critically ill patients despite the fact that the number of critically ill patients has increased recently. Since knowledge of the perceptions of family members about their needs and satisfaction is crucial in providing appropriate care for both family members and the patient, this study sought to explore such needs.

The study adopted the following key operational terms:

- *A critically-ill patient*: This is a patient admitted to ICU because of life threatening or potentially life-threatening physiological alterations.
- *Perceived need*: A need which is a requirement of family members and which if not met becomes a demand that may produce distress to the family members.
- *Level of satisfaction*: It is a measure of how services provided by a health facility meet clients’ expectations.
- *Services*: These are services provided by a health facility.,
- *Family member*: Is a partner or spouse, parent, grandparent, adult child, adult grandchild (older than 18 years), or identified adult significant other who visits the patient in the ICU.

## Methods

In this study a descriptive, cross sectional design was applied to assess the perceived needs and level of satisfaction with care provided by nurses. The study was conducted at Muhimbili National Hospital (MNH) in Dar es Salaam, which is one of the tertiary and large referral hospitals in Tanzania. It is a University Teaching Hospital with general and cardiac ICUs. The general ICU has 8 beds, while the cardiac ICU has 4 beds. More than 500 patients were admitted in both ICU units in 2009/2010 [8]. MNH was conveniently chosen for this study because it is among the tertiary hospitals where most critically ill patients and cardiac cases are admitted in Tanzania. Additionally, open heart surgery is performed in MNH.

### Sample size

The sample size was calculated using the formula: [*n* = 4σ2/ε2], where *n* = sample size, σ standard deviation, ε maximum likely error (0.59). The standard deviation was calculated after interviewing 16 family members, and was found to be 3.8. The total score for 15 individuals was calculated and found to be 52 scores, that is *n* = 4 × 3.8/0.59 × 0.59 = 165.9 making a total of 166 family members but a total of 110 family members of the critically-ill patients admitted in ICU in Muhimbili National Hospital were enrolled into the study between May and June 2010.

### Sampling procedure

The study units were selected through convenient sampling, guided by pre-set inclusion and exclusion criteria. Families of critically-ill patients admitted to ICU who could speak Swahili, male and/or female and of age 18 years and above were recruited in the study. Families who could not speak Swahili and under 18 years were excluded from this study. Family members visiting their relatives admitted to ICU for more than 24 h at the time of data collection were interviewed. At most, three relatives were interviewed for each patient. Eventually, a total of 110 family members of the critically-ill patients admitted to ICU in Muhimbili National Hospital were enrolled into the study between May and June 2010.

### Data collection

The data were collected from family members of critically-ill patients who were in ICU for more than 24 h using a questionnaire (see Additional file 1) which had been developed by researchers. The questionnaire was developed in English by the researchers and later translated into Kiswahili which is the national language and commonly spoken. All interviewed family members were conversant in Kiswahili. The questionnaire was made up of a demographic profile; perceived needs ranked in a Likert scale from 1 (not important) to 4 (very important); and questions about the level of satisfaction with care ranked on a Likert scale from 1 (no satisfaction) to 4 (complete satisfaction). Data was collected from participants who met the inclusion criteria of the study after seeking their consent.

### Pre-testing

Pre-testing was conducted with 16 family members of critically-ill patients admitted in the ICU. This was done to check if the instrument was able to collect valid information as desired and whether it was the appropriate length. Sixteen participants in the pilot study were not included in the main study. After the pre-testing of the questionnaire, unclear questions were modified or removed before actual data collection began.

### Validity

Content validity of the instrument was examined by three experienced intensive care nurses working in ICU and students doing their Masters degree in Critical Care and Trauma. After examining the questionnaire a discussion was held among the researchers to look into issues of clarity, specificity and length.

### Reliability

The test retest method was used to estimate the reliability of the instrument. This is the dependability with which an instrument measures an attribute or variable to establish if the participants in the study will be able to understand the instructions, the actual items, and respond appropriately to the instrument. The Cronbach’s alpha for the scale was 0.86 with standard deviation 0.88. This determines that the instrument was reliable [18]. Both Cronbach’s alpha and the standardized alpha values indicated a high degree of consistency for the scale, whereby the instrument is administered on the same participants twice and the correlation of their score is estimated. Response rates were high, exceeding 98 %. The high response rate could be due to the fact that the questionnaire was short (two pages) which was more acceptable for family members of ill patients in the hospital.

### Data analysis

Data was analyzed using descriptive and inferential statistics. The analysis was conducted using SPSS (version 14:0) software package. Frequency distributions of family members’ profiles mean scores for all items and associations among some characteristics were calculated using the same SPSS version.

### Ethical considerations

All participants were thoroughly informed about the study, their written consent was sought, and consent forms were signed upon agreement of participation. Confidentiality was maintained, as no names were used to identify participants; only numbers were used for identification. All rights of the participants such as freedom to participate or not, withdraw from the study, freedom of not answering some questions were addressed and observed. Ethical clearance was obtained from the Muhimbili University of Health and Allied Sciences (MUHAS) Research and Publications Committee. Permission to conduct the study at MNH was granted by the Muhimbili National Hospital.

## Results

### Demographic characteristics

Among the 110 study participants 51 (46.4 %) were males and 59 (53.6 %) were females. The mean age of the participants was 49.6 years. Out of the 110 participants, 12 (10.9 %) were parents of admitted critically-ill patients, 51 (46 .4 %) were brothers or sisters, 9 (8.2 %) were spouses, 10 (9.1 %) were children and 28 (25.4) were others. More than half (60 %) of the participants had primary school education or below, and 27.3 % had secondary school education. Post-secondary school education was found to be low, only 12.7 %. Table 1 shows the demographic profile of the participants.

**Table 1:** Demographic characteristics of family members of critically ill patients (*N* = 110)

Variables	Number (n)	Percent (%)
Gender		
Male	51	46.4
Female	59	53.6
Age		
18–27	21	19.1
28–37	39	35.5
38–47	27	24.5
48–57	16	14.5
58–67	6	5.5
68–77	1	0.9
Level of Education		
Primary or below	66	60
Secondary	30	27
College not degree	10	9.1
Degree	4	3.6
Relationship		
Parent	12	10.9
Brother/Sister	51	46.4
Spouse	9	8.2
Child	10	9.1
Others	28	25.4

### Needs of family members of critically ill patients

About three quarters of the participants 79 (71.8 %) perceived the need of having a specific person to call at the hospital when a family member is not available at the hospital as a very important need, while only 18.2 % of the respondents perceived this need as important.

The need to talk about the possibility of the patient dying was perceived as the least important need by 20.9 % of the participants; 22.7 % perceived this need as very important and 18.2 % said the need was important. The total percentage of the participants who perceived the need as not important was 38.2 %.

Table 2 illustrates the ten top important needs of family members of critically ill patients. The ten top important needs means ranged from 2.83 to 3.66. But Table 3 summarizes the least top ten needs that ranged from 2.26 to 2.80. The higher the arithmetic mean of the need, the more is the importance of the need. The least important need among the twenty needs was the need to talk about the possibility of the patient dying, with a mean score of 2.26.

**Table 2:** Summary of the nine most important family members’ needs (*N* = 110)

Rank in order of importance	Need	Mean score	SD
1	To have a specific person to call at the hospital in the absence of family member	3.66	.691
2	To be called at home about changes in the patient’s condition	3.61	.582
3	To see the patient frequently	3.61	.730
4	To have questions answered properly and explanations given in understandable terms	3.59	.691
5	To have someone concerned with family members’ worries	3.50	.776
6	To know how the patient is being treated	3.43	.760
7	To be assured that the best possible care and treatment are being given to the patient	3.30	.811
8	To receive information about the patient once per day	3.06	.964
9	To know about the type of staff taking care of the patient	2.95	1.092
10	To talk with the nurse each day	2.83	1.092

**Table 3:** Summary of the ten least important family members’ needs (*N* = 110)

Rank in order of less importance	Need	Mean score	SD
1	To talk about the possibility of the patient dying	2.26	1.215
2	To be told about transfer plans	2.47	1.190
3	To feel accepted by the hospital staff	2.47	1.070
4	To have directions regarding what to do at the bedside	2.48	1.190
5	To have friends nearby for support	2.58	1.049
6	To have explanation of the environment and machines around the patient	2.64	1.070
7	To help with the patient’s physical care	2.71	1.155
8	To have visiting hours or restrictions changed for special conditions	2.76	1.093
9	To talk with the doctor each day	2.79	1.112
10	To know the prognosis	2.80	1.152

### Association

Associations among the selected demographic characteristics using square tests were significant between age and need of having some explanation of the environment and machines around the patient (*x*^2^ = 26.46, df = 15, *P* < 0.05), as well as gender, and the need of having someone to explain what to do at the patient’s bedside (*x*^2^ = 9.1, df = 3, P < 0.05). Other associations which were statistically significant were level of education and prognosis (*x*^2^ = 17.82, df = 9, *P* < 0.05); level of education and need of receiving information about the patient once daily (*x*^2^ = 19.38, df = 9, *P* < 0.05); and level of education and the need of knowing the transfer plans (*x*^2^ = 18.85, df = 9, *P* < 0.05). The level of education and the need of knowing about the type of staff talking about the possibility of dying was not statistically significant (*x*^2^ = 22.54, df = 9, *P* < 0.01.

Relationship to the patient was statistically significant with the following needs: knowing how the patient was being treated (*x*^2^ = 17.58, df = 9, *P* = 0.05); knowing the prognosis (*x*^2^ = 17.87, *P* < 0.05), and talking with doctor each day (*x*^2^ = 17.22, df = 9, *p* < 0.05.

The findings indicated that more females perceived the need of being given explanation about what to do at the bedside as an important one. In total, 49 % of the females perceived this need as either important or very important compared to 44 % of the males who had the same views.

### Level of satisfaction of family members about care given at ICUs

Many of the respondents were satisfied with care given to the patients by nurses. Thirty point nine percent (30.9 %) were very satisfied; 42.7 % were satisfied compared to other levels of satisfaction about other types of care given to the family members of critically-ill patients.

Twenty one point eight percent (21.8 %) and 43.6 % of the respondents were very satisfied and satisfied respectively with ICU environment in general, while 34. 5 % were not satisfied

Mean score of level of satisfaction with care of family members of critically-ill patients ranged from 3.04 to 2.76. Level of satisfaction with care given to the patient by nurses scored the highest while the level of satisfaction with ICU environment in general scored the lowest. Associations among demographic characteristics using chi square test were significant between gender and the following levels of satisfaction: level of satisfaction with orientation about ICU (*x*^2^ = 9.37; df = 3, *P* < 0.05), level of satisfaction with the way the family members were cared for in the ICU (*x*^2^ = 9.45, df = 3, *P* < 0.005); and level of satisfaction with the communication between family members and staff about the patient’s condition. Other demographic characteristics showed no associations which were statistically significant. Relationship to the patient showed no statistically significant association with all levels of satisfaction with care.

## Discussion

Family members of critically ill patients serve as a bridge between care providers and a critically ill patient who is physiologically and psychologically compromised. To provide holistic care to the critically ill patient and their relatives, the needs and level of satisfaction with care of family members have to be known to health care providers.

### Needs of family members of critically Ill Patients

The need of having a specific person to call at the hospital when a family member is not available at the hospital was perceived as the highest by the family members, while the need of talking about the possibility of the patient dying was perceived the lowest. This was followed by the need of being called at home about changes in the patient’s condition, to see the patient frequently, having questions answered properly, and having someone to deal with family members’ concerns. These perceived needs ranked high by the family members, may be due to anxiety that their loved ones (patients) might die in their absence. In the African culture, family members/relatives prefer to be around their dying ones because last words by the dying are taken very seriously. This is probably because many people do not write wills, so they might give such important information during their dying moment.

Generally, information and assurance regarding needs of family members of critically ill patients needs to be given high priorities as indicated in previous study by Norris et al. [2]. Families wanted to know the patient’s current condition and progress. In addition, Day et al. [19] study findings indicated that the majority of family members of ICU patients experienced moderate to severe sleep (43.5 % out of 94 respondents) while 43.6 % experienced disturbance, fatigue and mild anxiety [19].

Families in this study needed to know how the patient was being treated and the type of staff who were taking care of the patient. Families required assurance that their patients were in safe hands.

In this study it has been observed in Tanzania that health providers do not normally communicate with the family member as required. This may be due to the belief that most family members do not necessarily have to know everything concerning their patient. About ten years back, most people in the country did not have cellular phones; they depended on landline phones which were scarce, mostly found in public and private offices and in a few well-to-do families. Currently, many Tanzanians own cellular phones and as a result the demand for communicating with health care providers regarding the condition of the patient has increased.

Most family members did not want to talk about the possibility of their family member dying. This is similar to other studies [1, 4, 14, 20] in which the family members saw the need of talking about the possibility of family members dying as not important. In the case of Tanzania, the need of family members not seeing the importance of talking about the possibility of their family members dying may be due to the trauma following one’s death, as death is taken to be a community responsibility unlike in developing countries. Also, it may be due to the myth that such talk might make the patient lose hope.

This study showed that the ten most important needs of family members of critically-ill patients were similar to other studies [1, 4, 9, 10], although ranking order differed slightly. For example, the eight most important family needs were to: have questions answered properly, know the prognosis, know why particular things were done to the patient, be called at home about change in patient’s condition, receive information daily, be assured that the best possible care was being given to the patient, be given explanation in understandable terms, and to feel that there was hope [9].

There was significant association between the need of being given explanation of the environment and machines around the patient. This could probably be attributed to the fact that many adults in the study had only primary education or less, so could not read explanations and other directions given in the ICU most of which are normally written in English. In Tanzania, English is the second language after Kiswahili and it is used as a medium of instruction starting from secondary level education. This finding does not correlate with previous studies [1, 21, 22].

There was also a significant association between gender and the need to have explanations about what to do at the patient’s bedside. Forty nine percent (49 %) of the 59 female respondents said it was important and very important to have explanation about what to do at the patient’s bedside compared to 44 % of males who had the same views. In Tanzania, the wife or mother is the one who takes care of the household especially when it comes to looking after children and ensuring good health of family members. This is probably why more females volunteered to assist critically-ill patients at the bedside during their visit, compared to males.

The results showed there was association between level of education and the need to know the prognosis, receive information daily, know the type of staff taking care of the patient, talk about the possibility of dying, and know the transfer plans. The results revealed that as the level of education increased the need also increased.

### Level of satisfaction of family members with care given in the ICU

The care given to the patients by nurses came first in the levels of satisfaction compared to other levels of satisfaction with other types of care given to the family members of critically-ill patients. Nurses are care givers who spend most of their time with patients and are the ones who meet with the families of critically-ill patients during their visits to the ICU. Their care can directly be assessed by these family members and hence the quality of such care could easily be determined.

In general, the ICU environment scored the least level of satisfaction among the ten items of care. There was no waiting room in the ICU for the family members during their visits. This could be one of the reasons why the family members were less satisfied with the ICU environment. This finding is different from findings from other studies done in Western countries where in most of the ICUs with waiting rooms for family members.

There was association between gender and level of satisfaction with orientation about ICU (P < 0.05), the level of satisfaction with the way the family members were cared in the ICU (P < 0.05), and level of satisfaction with the communication between family members and staff about the patient’s condition (P < 0.05). In all cases, male family members were more satisfied with the care received on these three items than females. No study has been documented to associate the same variables.

Relationship to the patient showed no statistically significant association with all levels of satisfaction with care. At this moment it is not easy to predict the reason why there was no association between relationship to the patient and level of satisfaction with care. No previous study could be found which has documented the same.

## Conclusions

The current study supports findings of many studies [1, 4, 14, 20] that have shown that most family members did not want to talk about the possibility of their family dying, as well as the ten most important needs of family members of critically-ill patients were similar to other studies [1, 4, 9, 10]. The findings from this current study provides useful evidence for policy makers and hospital managers in gaining understanding on what is required to meet the needs of the families of critically-ill patients.

## Additional file

Additional file 1: The Questionnaire on Perceived Needs and Level of Satisfaction with Care by Family Members of Critically Ill Patients at Muhimbili Intensive Care Units, Dar Es Salaam. (DOCX 27 kb)

## Abbreviations

ICU: Intensive Care Unit
MNH: Muhimbili National Hospital
