# Supplementary material for: Perceived needs and level of satisfaction with care by family members of critically ill patients at Muhimbili National hospital intensive care units, Tanzania
Source: BMC Nurs. 2016 Mar 9;15:18. doi: 10.1186/s12912-016-0139-5 (PMC4784468; doi:10.1186/s12912-016-0139-5)
Supplement: Additional file 1: — The Questionnaire on Perceived Needs and Level of Satisfaction with Care by Family Members of Critically Ill Patients at Muhimbili Intensive Care Units, Dar Es Salaam. (DOCX 27 kb) [file 12912_2016_139_MOESM1_ESM.docx]

**The Questionnaire on Perceived Needs and Level of Satisfaction with Care by Family Members of Critically Ill Patients at Muhimbili Intensive Care Units, Dar Es Salaam**

**Identification number:**……………….

1. **Demographic Data**
2. Age………………….years

**Circle the appropriate answer**

1. Sex:
2. Male
3. Female
4. Education background
5. Primary school and below
6. Secondary school
7. College (non- degree)
8. Degree
9. Religion
10. Christian
11. Islamic
12. Others, specify:……………………………………………
13. Relationship with patient
14. Parent
15. Brother/sister
16. Husband/wife
17. Others, specify:………………………………………………
18. **Needs of family Members**

Read the following items carefully to a family member and then put a tick in the appropriate box according to the family member’s perception about the need.

1. Means the need is not important
2. Means the need is less important
3. Means the need is important
4. Means the need is very important

Please indicate how the following aspects of critically ill patients’ care and family members’ care are important or unimportant to the family member.

| **SN** | **Item** | **1** | **2** | **3** | **4** |
| --- | --- | --- | --- | --- | --- |
| 1 | To know how the patient is being treated |  |  |  |  |
| 2 | To help with the patient’s physical care |  |  |  |  |
| 3 | To know the prognosis |  |  |  |  |
| 4 | To be called at home about changes in the patient’s condition |  |  |  |  |
| 5 | To receive information about the patient once per day |  |  |  |  |
| 6 | To know about the type of staff taking care of the patient |  |  |  |  |
| 7 | To be assured that the best possible care and treatment are being given to the patient |  |  |  |  |
| 8 | To have questions answered properly and explanations given in an understandable terms |  |  |  |  |
| 9 | To have specific person to call at the hospital when not there |  |  |  |  |
| 10 | To talk about the possibility of the patient’s death |  |  |  |  |
| 11 | To be told about transfer plans |  |  |  |  |
| 12 | To have directions regarding what to do at the bedside |  |  |  |  |
| 13 | To see the patient frequently |  |  |  |  |
| 14 | To feel accepted by the hospital staff |  |  |  |  |
| 15 | To have visiting hours or restrictions changed for special conditions |  |  |  |  |
| 16 | To have someone concerned with the family members’ concerns |  |  |  |  |
| 17 | To talk with the doctor each day |  |  |  |  |
| 18 | To talk with the nurse each day |  |  |  |  |
| 19 | To have friends nearby for support |  |  |  |  |
| 20 | To have explanations of the environment and machines around the patient |  |  |  |  |

1. **Level of Satisfaction of Care**

Read the following items carefully to a family member and then put a tick in the appropriate box according to the family member’s level of satisfaction with care.

Level 1 means not satisfied with care

Level 2 means less satisfied with care

Level 3 means satisfied with care

Level 4 means very satisfied with care

Please indicate the level of satisfaction of family members with care given to their critically ill patients

| **SN** | **Item** | **1** | **2** | **3** | **4** |
| --- | --- | --- | --- | --- | --- |
| 1 | To what level are you satisfied with the reception you got in ICU? |  |  |  |  |
| 2 | To what level are you satisfied with orientation given to you about the ICU? |  |  |  |  |
| 3 | To what level are you satisfied with the information given to you about the patient’s progress? |  |  |  |  |
| 4 | To what level are you satisfied with the care given to your patient by ICU nurses? |  |  |  |  |
| 5 | To what level are you satisfied with the care given to your patient by the doctors? |  |  |  |  |
| 6 | To what level are you satisfied with the visiting hours? |  |  |  |  |
| 7 | To what level are you satisfied with the way you were cared for in the ICU? |  |  |  |  |
| 8 | To what level are you satisfied with the communication between you and ICU staff about the patient’s condition? |  |  |  |  |
| 9 | To what level are you satisfied with the ICU environment in general? |  |  |  |  |
| 10 | To what level are you satisfied with other family members are cared by ICU staff? |  |  |  |  |
